# The Problem of the Discharged Soldier

**Published:** 1918-09-14

**Authors:** 


### THE LONDON HOSPITAL QUARTERLY COURT.

### The Problem of the Discharged Soldier.

THE report presented to the Court on September 4 states that since the last report the Cavell Home for Nurses has become occupied. There was no formal opening, the nurses simply moved in. For the furnishing of the beautiful sitting-room of this Home many gifts were received. The bust of Edith Cavell was presented by the sculptor, Sir George Frampton, R.A., who also collected from his private friends gifts of beautiful engravings for the decoration of the walls. Mr. Foster, a member of the Committee, has also given some very fine reproductions from the old masters for the decoration of this room.

### LADY ALMONERS AND THE MARIE CELESTE SOCIETY.

Hitherto the work of the lady almoners, which is associated with the out-patient department, has been carried on separately from the Marie Celeste Samaritan Society of the hospital, a society that has been in existence for more than one hundred years, but which only deals with the in-patients.

It seems right, both to the House Committee and to the Samaritan Society, to organise an amalgamation and so to prevent the overlapping of duties and duplication of cost. After much consideration this has been arranged. The hospital, instead of paying the cost of the almoners direct, will pay £1,500 a year to the Samaritan Society, and that society will "take over" the out-patients' almoners.

### NURSES AND THE PARLIAMENTARY FRANCHISE.

The hospital has been informed by the Registration Officer of the district that the nurses, who occupy each a separate bedroom, are entitled to have their names entered on the register and to vote both in borough and Parliamentary elections; therefore full returns of the nursing staff who fulfilled the conditions as to age and length of residence have been supplied in addition to returns of the resident medical officers.

### DISCHARGED SAILORS AND SOLDIERS.

The Ministry of Pensions has arranged, as a temporary measure, for treatment of discharged sailors and soldiers to be supplied by the voluntary hospitals, as they alone have the medical and surgical staff required, and the complicated and expensive equipment also needed. The Ministry pays the hospital at the rate of 2s. for each attendance if the patient is treated as an out-patient, and 7s. per day if admitted to the wards as an in-patient. The scheme is very easily stated on paper, but has not been very easy to work efficiently.

When a man is discharged from the Navy or Army he is given a card to take to one or other of the Pensions Committees—the nearest to his home. There are about fifty of such Pensions Committees in London alone. If the man requires further treatment he applies to one of these local committees and is given an order to come to the hospital for treatment. Then the difficulties begin. The first is that he brings no notes of his former treatment. He may have had several previous operations, and may be complaining of pain, but the hospital does not know what they were and so cannot guess what may be the cause of the pain; or a nerve may have been cut by a piece of shrapnel and it is impossible to tell whether it has been sutured or not. Then there is the difficulty in getting tuberculous cases, of which there seem to be a great number, treated.

The length of treatment required by certain classes of cases has been a trouble. Some forms of nerve injury may occupy a bed for many months; the beds are gradually filled with these long-time cases, and so cease to be of the same value, as a hospital is intended to treat acute illness. The average time of residence of ordinary patients is about twenty days. A surgeon with, say, twelve beds, and who gets one or two of these nerve cases, finds himself unable to cope with the ordinary cases waiting to come in, and so the "waiting-list" mounts up.

Sometimes the hospital is put in a difficult position because the physician or surgeon entirely disagrees with the discharge of the man from the Army at all. He has been discharged for heart disease, for instance. No sign of disease can be found, and when the eternal cigarette habit has been stopped the symptoms quite disappear.

### Quinquennial Appeal.

The appeal for the million half-crowns is doing well. The amount hitherto collected for the quinquennial appeal is £107,664—*i.e.* 861,311 half-crowns. Mr. George Robey has been made a life governor, the only means of acknowledging the great help received from him in opening a special appeal for the hospital at Messrs. Selfridge's, to whom also sincere thanks are extended.

### Women Students.

Women students are to be admitted to the hospital, and the session will open in October. The committee have sanctioned the Medical College spending a sum of £887 in certain necessary alterations in class-rooms and cloak-rooms, etc.

### Difficulties in Working the Hospital.

The work of the hospital is still carried on with great difficulty, and there is not only the shortage of surgeons, physicians, resident doctors, nurses, servants, and porters to contend with, but there is also the increased work which is dependent upon the various rationing Orders.

